# Impact of Co-Presence of Endotoxins and Microplastics on Seawater Biophysicochemical Indicators

**DOI:** 10.3390/ijms26157178

**Published:** 2025-07-25

**Authors:** Hasan Saygin, Asli Baysal

**Affiliations:** 1 Application and Research Center for Advanced Studies, Istanbul Aydin University, Sefakoy Kucukcekmece, Istanbul 34295, Türkiye; 2 Department of Chemistry, Faculty of Science and Letters, Istanbul Technical University, Istanbul 34469, Türkiye; asli.baysal@itu.edu.tr

**Keywords:** plastics, marine ecosystem, bacteria, leaching, biofouling

## Abstract

Micro/nanoplastics (MNP) and endotoxin, typical emerging contaminants, can be found in marine aqueous systems due to various natural and anthropogenic activities, and their co-occurrence may influence the biophysicochemical characteristics of seawater. Moreover, endotoxins may be transported by the micro/nanoplastics or increase the deformation of these substances, comprising other risks to the ecosystem. However, the impacts of the co-occurrence of micro/nanoplastics and endotoxins in seawater remain unknown. We studied the effects of endotoxin at three concentration levels in seawater and its combined impact with micro/nanoplastics at three doses on biophysicochemical processes in seawater through spectroscopic analysis, leaching indicators (turbidity and humidification index), oxidative potential, antioxidant activity, and biofilm production. The results showed that the UV–VIS spectra of seawater changed with their co-occurrence. The co-presence of MNPs and endotoxins increased the turbidity in seawater, indicating the leaching of micro/nanoplastic in the presence of endotoxins. A higher humification index in seawater showed the formation of dissolved organic substances in micro/nanoplastic and endotoxin seawater compared to the results for untreated seawater. Dithioerythritol assay revealed the differences in oxidative potentials of plain seawater and seawater in the co-presence of micro/nanoplastics and endotoxins. An important biochemical reaction in seawater was tested using biofilm formation. The results showed higher biofilm formation in their co-presence. This study provides new insights into the effects of micro/nanoplastics and their composite pollution with endotoxins on biophysiochemical indicators in seawater.

## 1. Introduction

Marine aqueous environments, whether they are in natural, recreational, or industrial areas, play critical roles in biogeochemical processes and human health. The quality, e.g., physicochemical and biological characteristics, and availability of marine aqueous environments fluctuate because of the impact of natural and human contributors [1,2,3,4].

Plastic pollution is an emerging environmental problem owing to its widespread use and improper disposal [5]. Under natural conditions including marine aqueous environments, plastic products can easily degrade into smaller particles called micro/nanoplastics (MNPs), and they are a pressing environmental issue [6]. In addition to the ecotoxicity of the individual presence of MNPs, they also sorb, transport, and leach the substances that show significant environmental stressors [1,3,5,7]. For example, MNPs are known to leach their organic substances, including phthalates, dissolved organics, and metals, in aqueous environments [4,8]. However, their leaching under various conditions has been limitedly examined. It is also known that MNPs can interact with various biological or chemical substances, including proteins, metals, and microorganisms, and these substances can be loaded onto the surface of MNPs [9,10,11].

Another important environmental stressor on marine aqueous environments is microbial pollution. Various sources, such as sewage discharge and agricultural runoff, can contribute to microbial pollution. This poses a substantial risk to the environment and public health [12]. Microbial biomass, fecal contamination, and aqueous environmental quality can be identified by the presence of endotoxins [13]. Since endotoxins are the complex substances of lipopolysaccharide found in the outer membrane of Gram-negative bacteria and some cyanobacteria and are available in various environmental components, they are accepted as one of the emerging pollutants in the environment and are of immediate concern in many environmental components, including seawater, air, and industrial processes [12,13,14]. They are heat- and pH-resistant due to their amphoteric nature, contain three different chemical characteristics, and can also leak into aqueous environments during the growth and proliferation of the bacteria [14]. Furthermore, endotoxins can be formed through by-products from dead bacteria, producing a fever-inducing substance, along with inflammation factors [14,15]. Therefore, they are known to have an adverse health impact on living systems, including humans [14,15].

Moreover, the presence of microbial biomass in aqueous systems may trigger degradation of MNPs, and their co-presence may cause more harmful effects to the environment. For example, the leached substances, owing to the microbial deformation of MNPs, can promote the colonization of microbial components, dissolved organic substances from MNPs can influence the medium physicochemical characteristics and biogeochemical reactions during the interaction between MNPs and microbial biomass, or microbial biomass can attach to the MNPs and be transported along with them [1,4,6,16,17].

Therefore, our study aims to examine the co-presence of endotoxin and MNPs in seawater through multiple biophysicochemical indicators. Changes in seawater owing to co-exposure of various concentrations of endotoxins and different doses of MNPs were characterized by spectroscopic analysis, leaching characterization (turbidity and humidification indices (HI)), oxidative potential, antioxidant activity, and biofilm formation.

## 2. Results

**Figure 1:**
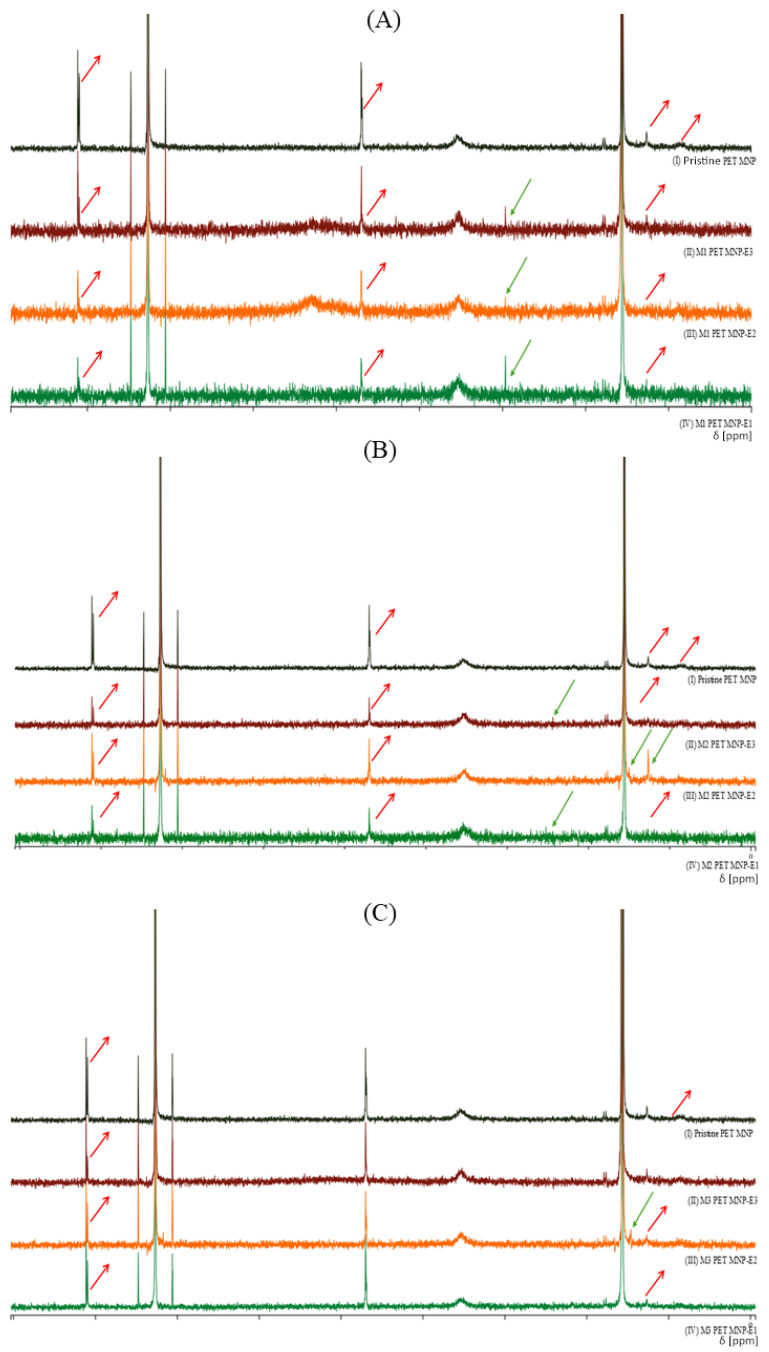
H-NMR spectra of PET MNPs before and after endotoxin exposures at three concentrations in seawater. (**A**) PET MNP at the dose of 1 mg/mL before and after endotoxin exposures at three concentrations, (**B**) PET MNP at the dose of 10 mg/mL before and after endotoxin exposures at three concentrations, and (**C**) PET MNP at the dose of 30 mg/mL before and after endotoxin exposures at three concentrations. Black line: pristine form of PET MNPs; green line: 0.10 EU/mL of endotoxin; orange line: 0.50 EU/mL of endotoxin; dark red line: 2.00 EU/mL of endotoxin. Green arrow: Formation of a new signal or increased intensity, Red arrow: Removal or reduction of signals.

The characterization of biophysicochemical indicators of seawater was assessed through various parameters such as MNP structural changes and seawater characteristics. Firstly, as seen in Figure 1, the interaction between MNP and endotoxin on the PET MNP structures was characterized using H-NMR. The spectra showed the removal and reduction of some PET characteristic signals, such as peaks at δ 1.01 and 8.11 ppm, which were assigned to CH and CH_3_, respectively, and aromatic protons and CH_2_ close to the ester.

Table 1 shows the endotoxin retention after PET MNP treatment in seawater. The results indicated that the highest retention (24.5 ± 3.3%–57.1 ± 2.6%) was observed after the lowest MNP treatments compared to the results for the medium (2.3 ± 4.1%–9.1 ± 2.5%) and highest MNP (3.2 ± 1.8%–8.6 ± 2.3%) doses. Moreover, the highest level of endotoxin retention by MNP was achieved at the lowest endotoxin concentration and with the lowest MNP dose. However, increases in endotoxin concentrations resulted in higher levels of endotoxin retention with increasing MNP doses.

**Figure 2:**
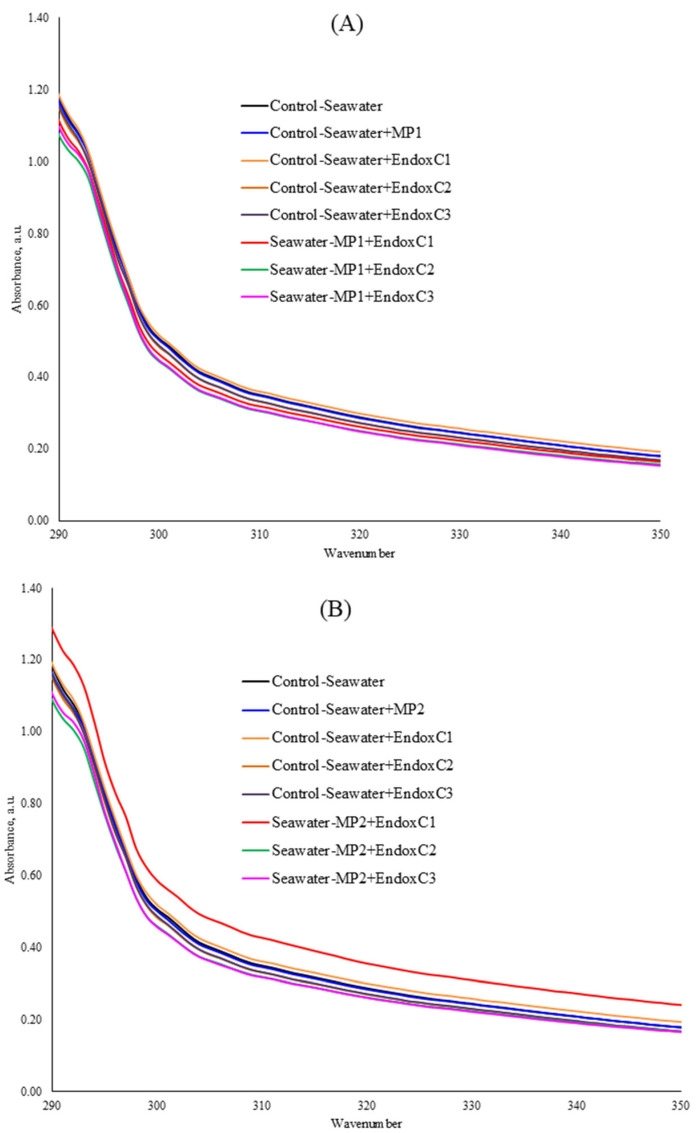

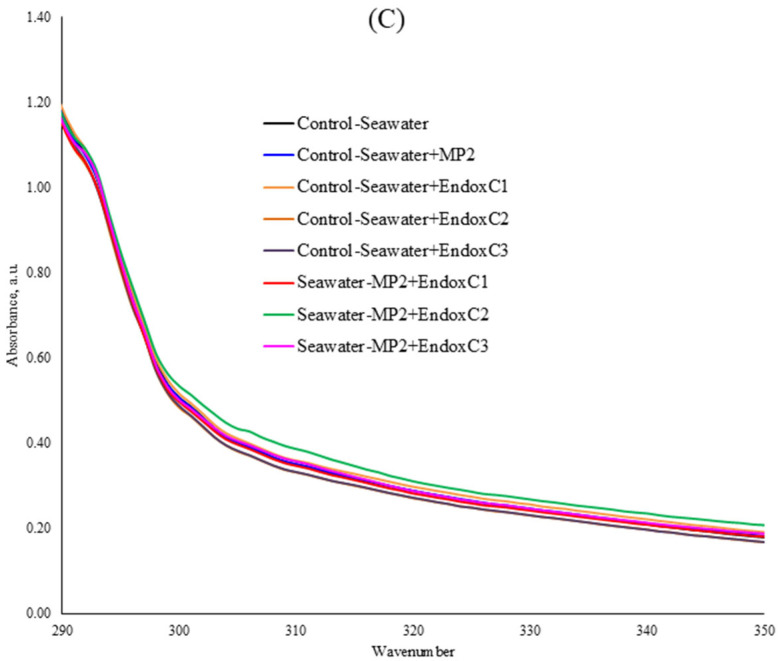
UV–VIS spectra of seawater before and after MNP and endotoxin exposures. (**A**) Seawater before and after PET MNP at the dose of 1 mg/mL (MP1) and endotoxin exposures at three concentrations, (**B**) seawater before and after PET MNP at the dose of 10 mg/mL (MP2) and endotoxin exposures at three concentrations, and (**C**) seawater before and after PET MNP at the dose of 30 mg/mL (MP3) and endotoxin exposures at three concentrations. EndoxC1: 0.10 EU/mL of endotoxin; EndoxC2: 0.50 EU/mL of endotoxin; EndoxC3: 2.00 EU/mL of endotoxin.

Figure 2 shows the UV–VIS absorption spectra of seawater in the presence of MNPs and endotoxin. The UV–VIS spectrum of the seawater after treatment with MNPs and endotoxin indicated that the spectrum lines regularly changed owing to the interaction of seawater with the MNPs and endotoxin. The absorption peak at 306 nm was recorded to observe these changes specifically (Table 2). For instance, at the lowest MNP exposure dose, the spectra of seawater significantly declined in the co-presence of MNPs and different concentrations of endotoxin at the levels of 9–13% (*p* < 0.05). At the medium MNP exposure dose, the addition of the lowest concentration of endotoxin significantly increased the UV–VIS absorption compared to that of the control seawater; however, the absorbances significantly declined with increasing endotoxin concentrations without MNP contamination. Similarly, lower absorbance values (9–11%) were observed in the co-presence of a medium dose of MNP and medium and higher concentrations of endotoxin, rather than the lowest endotoxin concentration (significantly higher absorbance at the lowest endotoxin and medium MNP dose at the level of 20%), indicating the MNP dissolution. In contrast, at the highest MNP dose, the addition of a low concentration of endotoxin insignificantly reduced the UV–VIS absorption of seawater at the level of 1%, which then increased with increasing endotoxin concentrations. Although this higher absorbance was significant in the co-presence of the medium endotoxin concentration and the highest MNP dose (*p* < 0.05), it was not significant at the highest endotoxin concentration with this MNP dose.

**Figure 3:**
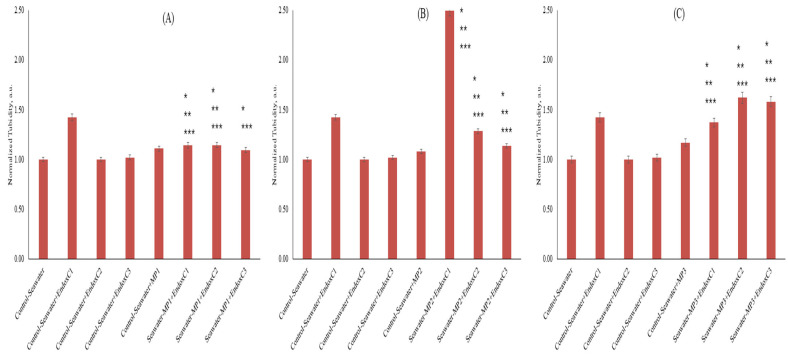
Turbidity of seawater before and after MNP and endotoxin exposures. (**A**) Seawater before and after PET MNP at the dose of 1 mg/mL (MP1) and endotoxin exposures at three concentrations, (**B**) seawater before and after PET MNP at the dose of 10 mg/mL (MP2) and endotoxin exposures at three concentrations, and (**C**) seawater before and after PET MNP at the dose of 30 mg/mL (MP3) and endotoxin exposures at three concentrations. EndoxC1: 0.10 EU/mL of endotoxin; EndoxC2: 0.50 EU/mL of endotoxin; EndoxC3: 2.00 EU/mL of endotoxin. (*) indicates significant difference compared to control-seawater; (**) indicates significant difference compared to control-MP + seawater; (***) indicates significant difference compared to control-Endox + seawater at the same endotoxin concentration.

To understand the co-exposure impact of MNPs and endotoxins on seawater via the agglomeration state, the turbidity was measured in the seawater samples (Figure 3). The results showed significantly higher turbidity (43%) in the presence of low concentrations of endotoxin (*p* < 0.05). However, turbidity levels decreased as endotoxin concentrations increased. No differences were observed in turbidity, which was kept below 2%, at medium and higher concentrations of endotoxin compared to the results for control seawater. On the other hand, turbidity increased at the levels of approximately 8–17% in the presence of MNP with increasing doses. Furthermore, the results showed that the co-presence of MNPs and endotoxins significantly influenced turbidity in seawater compared to the results for untreated seawater and the individual presence of MNPs and endotoxins. For example, at the low dose of MNP exposure, approximately 15% higher turbidities were obtained at the low and medium concentrations of endotoxin, whereas it was approximately 10% at the highest endotoxin concentration. At the medium dose of MNP exposure, more significant and higher turbidity levels were measured (*p* < 0.05). There was also a reduced trend of endotoxin concentration. At the highest MNP dose, significantly higher turbidity levels (37–62%) were found with increasing endotoxin concentrations (*p* < 0.05). Moreover, with the increasing MNP dose, the level of turbidity of seawater declined with increasing endotoxin concentrations.

**Figure 4:**
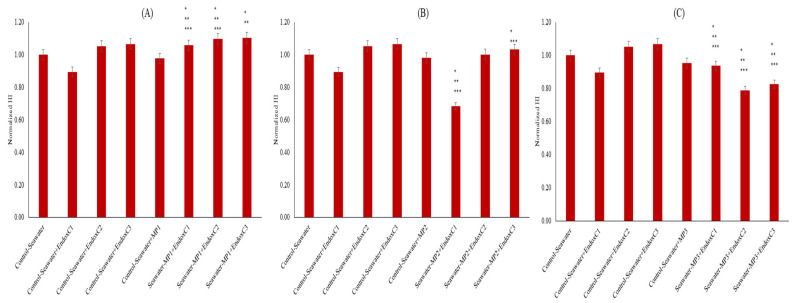
HI of seawater before and after MNP and endotoxin exposures. (**A**) Seawater before and after PET MNP at the dose of 1 mg/mL (MP1) and endotoxin exposures at three concentrations, (**B**) seawater before and after PET MNP at the dose of 10 mg/mL (MP2) and endotoxin exposures at three concentrations, and (**C**) seawater before and after PET MNP at the dose of 30 mg/mL (MP3) and endotoxin exposures at three concentrations. EndoxC1: 0.10 EU/mL of endotoxin; EndoxC2: 0.50 EU/mL of endotoxin; EndoxC3: 2.00 EU/mL of endotoxin. (*) indicates significant difference compared to control-seawater; (**) indicates significant difference compared to control-MP + seawater; (***) indicates significant difference compared to control-Endox + seawater at the same endotoxin concentration.

Figure 4 illustrates the normalized levels of HI. The HI level was lower at the lowest concentration of endotoxin compared to the results for the control of seawater (11% lower HI level); however, it was increased with higher concentrations of endotoxin (approximately 5–7%). Similarly, lower HI levels (approximately 2–5%) were measured when seawater was treated with the individual presence of MNPs. Moreover, HI levels changed significantly when seawater was exposed to endotoxins and MNPs together (*p* < 0.05), except at the medium dose of MNPs and the medium endotoxin concentration. There was an increasing trend with endotoxin concentration at the lowest and medium MNP dose, whereas there was a declining trend with endotoxin concentration at the highest MNP dose.

**Figure 5:**
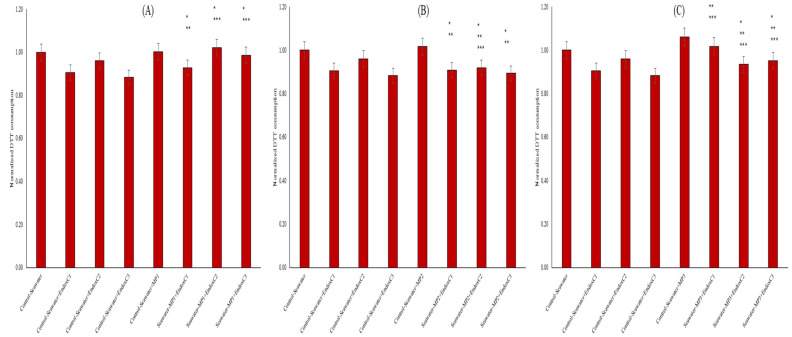
OP by DTT assay of seawater before and after MNP and endotoxin exposures. (**A**) Seawater before and after PET MNP at the dose of 1 mg/mL (MP1) and endotoxin exposures at three concentrations, (**B**) seawater before and after PET MNP at the dose of 10 mg/mL (MP2) and endotoxin exposures at three concentrations, and (**C**) seawater before and after PET MNP at the dose of 30 mg/mL (MP3) and endotoxin exposures at three concentrations. EndoxC1: 0.10 EU/mL of endotoxin; EndoxC2: 0.50 EU/mL of endotoxin; EndoxC3: 2.00 EU/mL of endotoxin. (*) indicates significant difference compared to control-seawater; (**) indicates significant difference compared to control-MP + seawater; (***) indicates significant difference compared to control-Endox + seawater at the same endotoxin concentration.

Another vital parameter for the biochemical processes in seawater includes the oxidative characteristics. The OP results revealed the lower OP levels in seawater after treatments compared to those for the untreated seawater (Figure 5). The presence of individual MNPs increased the OP of seawater in a dose-dependent manner. For example, there was no impact at a low concentration of endotoxin, whereas there was an impact at medium and high concentrations, at 2% and 7%, respectively. However, the individual presence of endotoxin significantly reduced the OP of seawater at the levels of approximately 6–12% (*p* < 0.05). Furthermore, variable effects were observed when endotoxin and MNP were found together. For example, at the lowest MNP exposures, their co-presence in seawater increased the OP levels when combined with endotoxin concentrations compared to the levels found for the individual presence of endotoxin, and the differences were significant at the medium and highest endotoxin concentrations (*p* < 0.05). Conversely, at medium MNP dose, OP levels declined, and insignificant changes were found compared to the results for the control at the same endotoxin concentrations. At the highest MNP exposures, higher OP levels declined with increasing endotoxin concentrations. This was significant in the control with the same endotoxin concentration (*p* < 0.05).

**Figure 6:**
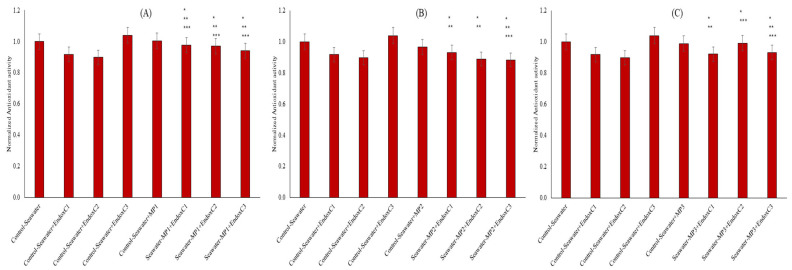
Antioxidant activity of seawater before and after MNP and endotoxin exposures. (**A**) Seawater before and after PET MNP at the dose of 1 mg/mL (MP1) and endotoxin exposures at three concentrations, (**B**) seawater before and after PET MNP at the dose of 10 mg/mL (MP2) and endotoxin exposures at three concentrations, and (**C**) seawater before and after PET MNP at the dose of 30 mg/mL (MP3) and endotoxin exposures at three concentrations. EndoxC1: 0.10 EU/mL of endotoxin; EndoxC2: 0.50 EU/mL of endotoxin; EndoxC3: 2.00 EU/mL of endotoxin. (*) indicates significant differences compared to control-seawater; (**) indicates significant differences compared to control-MP + seawater; (***) indicates significant difference compared to control-Endox + seawater at the same endotoxin concentration.

The antioxidant capacity in seawater was assessed by CUPRAC assay to understand the oxidative processes (Figure 6). The presence of individual endotoxins decreased the antioxidant capacity of seawater by 8–10%, except at the highest concentration. Similarly, the presence of MNPs limitedly reduced the antioxidant capacity of seawater. No change to 3% reduction in antioxidant activities was measured with individual MNP contamination of seawater. The results reveal that the co-presence of endotoxins and MNPs also influenced the antioxidant activity in seawater. For example, at the low MNP dose, their co-presence increased the antioxidant activities of seawater when compared to the results for the individual presence of endotoxin at low and medium concentration of endotoxins, but it was significantly lower at the highest endotoxin concentration (*p* < 0.05). At medium-dose MNP exposures, mainly significantly lower antioxidant levels at the low and medium endotoxin concentrations were obtained when compared to results for control seawater and the individual presence of MNPs, whereas it was significantly lower at the highest endotoxin concentration (approx. 12%). Similarly, lower antioxidant activities (6–8%) were found in the co-presence of the highest dose of MNP and low and high endotoxin concentrations compared to the results for seawater control (*p* < 0.05). Moreover, increased MNP doses and endotoxin concentration decreased the antioxidant activity in seawater when they were found together in seawater.

**Figure 7:**
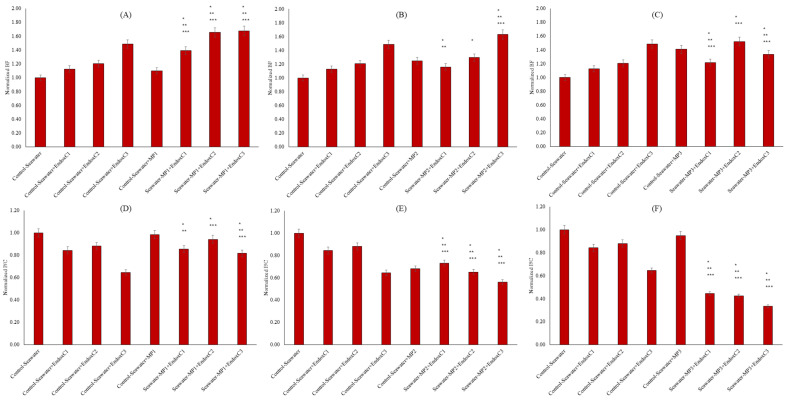
Biofilm formation and P/C of seawater before and after MNP and endotoxin exposures. (**A**) Biofilm formation of seawater before and after PET MNP at the dose of 1 mg/mL (MP1) and endotoxin exposures at three concentrations, (**B**) biofilm formation of seawater and after PET MNP at the dose of 10 mg/mL (MP2) and endotoxin exposures at three concentrations, and (**C**) biofilm formation of seawater before and after PET MNP at the dose of 30 mg/mL (MP3) and endotoxin exposures at three concentrations. (**D**) P/C of seawater before and after PET MNP at the dose of 1 mg/mL (MP1) and endotoxin exposures at three concentrations; (**E**) P/C of seawater and after PET MNP at the dose of 10 mg/mL (MP2) and endotoxin exposures at three concentrations, and (**F**) P/C of seawater before and after PET MNP at the dose of 30 mg/mL (MP3) and endotoxin exposures at three concentrations. EndoxC1: 0.10 EU/mL of endotoxin; EndoxC2: 0.50 EU/mL of endotoxin; EndoxC3: 2.00 EU/mL of endotoxin. (*) indicates significant difference compared to control-seawater; (**) indicates significant difference compared to control-MP+seawater; (***) indicates significant difference compared to control-Endox + seawater at the same endotoxin concentration.

As illustrated in Figure 7A–C, the presence of endotoxin significantly increased the colonization in seawater in a dose-dependent manner compared to the results for seawater control (12%, 21% and 49% at the low, medium, and high endotoxin concentrations, respectively) (*p* < 0.05). Similarly, individual contamination of MNP in seawater promoted biofilm production at the levels of 10%, 25%, and 41% for low, medium, and high doses of MNP, respectively. These changes were significant at the level of *p* < 0.05. Furthermore, the co-presence of MNPs and endotoxins increased the biofilm formation in seawater compared to that in untreated seawater, the individual presence of MNPs in seawater, and the individual presence of endotoxins. In the low and high MNP exposures, these higher biofilm productions were significantly different when compared to those in seawater control, the individual presence of MNPs in seawater, and the individual presence of endotoxins (*p* < 0.05). To understand the chemical background of biofilm formation, a correlation analysis was conducted, and the results indicated the significant importance of HI and OP in biofilm formation. For example, at the low and medium doses of MNP, the co-presence with endotoxin was positively linked to seawater biofilm production and HI (r = 0.98 and r = 0.77 for the low and medium MNP, respectively). Similarly, a positive correlation was found between OP and biofilm production in the co-presence of endotoxin and at the low dose of MNP (r = 0.90). In addition to biofilm formation quantity, the chemical characteristic of biofilms was assessed by protein to carbohydrate ratio (P/C). The P/C levels are presented in Figure 7D–F. The individual presence of MNPs showed an insignificantly lower P/C ratio at the low and high MNP doses (*p* > 0.05), but it was significantly lower at the medium MNP dose (approx. 32% reduction) (*p* < 0.05), indicating acidic properties of the biofilms. Similarly, significantly lower P/C ratios were observed with individual contamination of endotoxin when compared with those in seawater control (*p* < 0.05). The reductions were at the level of approximately 16%, 12%, and 36% in the low, medium, and high concentrations of endotoxin, respectively. When seawater was contaminated with MNP and endotoxin together, the P/C ratios were decreased. Synergistically, the data showed a reduction in the P/C ratio with increasing endotoxin and MNP levels. The findings were significant with increasing MNP doses when compared to all conditions, e.g., seawater control, individual doses of MNP, and individual endotoxin concentrations (*p* < 0.05).

## 3. Discussion

According to H-NMR spectra (Figure 1), the introduction of new peaks in PET MNPs with endotoxin exposures in seawater indicated the attachment of methoxy groups, b-hydroxy fatty acids, or other aliphatic linkages owing to the endotoxin [18]. Moreover, the introduction or removal of signals revealed the leaching of MNPs with endotoxin, as well as interaction between MNPs and endotoxin [19,20]. Table 1 indicates the endotoxin retention after MNP treatments. These results are also in good agreement with the H-NMR spectra. However, the reduction in the endotoxin retention with MNP doses can be explained by the sorption and desorption properties and surface deformation of the MNPs [1,3,5,7].

As seen in Figure 2 and Table 2, the reduction in UV–VIS absorbances may originate from MNP leaching [21]. Various studies explained the loss in UV–VIS absorbance as an indicator of particle deformation or dissolution [21]. Furthermore, the presence of MNPs and endotoxin in seawater can result in higher UV–VIS absorption spectra due to complexation.

Since turbidity is an indicator of released substances after leaching [2], the higher turbidity results revealed the leaching of MNPs with endotoxins in seawater (Figure 3) [1,2,5,8]. Although the leaching of the MNPs in various media was previously examined in a limited number of studies [1,2,5,8], Luo et al. [7] stated that the weathering of MNPs also caused the release of pigments in simulated fluids, and the higher turbidity revealed the binding between the released pigments and enzymes. However, the reduction in turbidity with increasing endotoxin concentrations can originate from the protection of the MNP leaching via covering with the increasing endotoxin concentration because some studies revealed the covering of the MNP surfaces with different biomolecules [22,23].

HI is an indicator of changes in organic solute aromaticity, and humification can be used to identify the changes in the composition of organic substances in the environmental media, which influence the biochemical processes [17,24]. The increasing HI levels of seawater with the co-presence of MNPs and endotoxins compared to the levels for untreated seawater can be explained by the formation of dissolved organic substances in seawater with MNPs and endotoxins (Figure 4). However, when their co-presence is considered, the reduction in HI with increasing MNP doses can be attributed to complexation between MNPs and endotoxins, with the protection effect of endotoxins exerted on the MNP leaching [25,26].

Deformation or leaching of MNPs in the seawater with the presence of endotoxin was also evaluated via OP levels (Figure 5). The lower OP results with the exposure of endotoxin can be explained by the scavenging of oxidative substances with endotoxin, since endotoxins play a role in the secretion of the molecules [27]. The higher OP levels of seawater with the individual MNP exposures can be attributed to the substances released from MNPs to seawater. On the other hand, the increasing OP levels with the co-exposure of MNP and endotoxin may show that the impact of the individual presence of endotoxin on the secretion of radical molecules may be disturbed by the addition of MNPs.

Since antioxidant capacity plays a critical role in examining the biochemical responses of the medium and substances to environmental factors, it is also used to assess the tolerance of the medium in the face of natural stressors [28]. It is known that antioxidant activity can be influenced by medium components, including the formation of reactive species [29]. As illustrated in Figure 6, the reduction in antioxidant capacities may originate from the increased oxidative species in seawater owing to the co-presence of MNPs and endotoxins, along with the higher leachability of MNPs with endotoxin concentration.

The bioactivity of seawater was tested using biofilm formation (Figure 7). Higher biofilm formation can be explained by the leaching of dissolved organic substances from MNPs with their co-exposure to endotoxin. Dissolved organic substances mainly determine biofilm production, and dissolved organic substances seem to be more influential than other factors, such as surface properties [9]. Moreover, the reduction in HIs and higher OPs can contribute as environmental stressors, and microorganisms may form biofilm to protect themselves owing to lower HI and higher OP levels in seawater. Thus, biofilm formation is an important issue for the marine environment. The triggering effect of the co-presence of these contaminants is another issue and needs to be examined [16]. Biofilm characteristics were evaluated by P/C, as illustrated in Figure 7D,F. Since microbial polymeric substances contain various molecules, including proteins and carbohydrates, the aggregation of these molecules dominates the surface hydrophobicity and hydrophilicity [30,31,32,33]. For instance, proteins can be characterized as hydrophobic, and carbohydrates exhibit hydrophilic characteristics. Thus, the dominant characteristic of the conditions in the biofilm production significantly influences the surface processes, including surface activity, stickiness, attachment, or aggregation probability [32,33]. A lower P/C ratio indicates the formation of acidophilic biofilms. This finding may cause higher affinity for alkaline rather than acidic substances [30,31].

Overall, the results showed that the co-presence of endotoxins and MNPs exerted an antagonistic effect on the HI and antioxidant activities of seawater, with a synergistic impact on its turbidity. This finding suggests that organic substances can be scavenged by the endotoxins and that the leaching of MNPs can increase oxidative species. Furthermore, the formation of biofilms, as well as the acidic background of the biofilms, can be explained by the combined effect of the presence of endotoxins and MNPs, which exert a negative effect on HI and antioxidant activities.

## 4. Materials and Methods

In this study, a commercially available plastic product (plastic drinking water bottles) was used throughout the experiments. The product was purchased from the local market, and the brand of product was widely found in all levels of markets in Turkey. To produce micro-sized plastics, the in-house method was applied based on our previous studies [34,35]. Briefly, the plastic water bottles were thoroughly washed with ultrapure water (Milli-Q, Merck, Darmstadt, Germany) and dried in the clean room facilities. Then, cleaned bottles were shredded with a stainless-steel render and sieved to create small particles. The structural and chemical properties were previously characterized using attenuated total reflectance Fourier transform infrared spectrometry (ATR-FTIR, Bruker InvenioS, Billerica, MA, USA), scanning electron microscopy with energy-dispersive x-ray spectroscopy (SEM–EDX, FEI, Thermo Fisher Scientific, Waltham, MA, USA), DLS (Zetasizer Nano ZS, Malvern Instruments, Malvern, UK), Brunauer–Emmett–Teller surface area by multipoint measurement (BET, Micromeritics Gemini VII 2390 t, Norcross, GA, USA), and transmission electron microscopy (TEM, Hitachi HighTech HT7700, Minato, Japan), and the characterization results were presented in the study of Baysal et al. [36].

For the exposure process, we conducted ex situ experiments. Seawater was collected from Marmara Sea, Istanbul, Turkey. To obtain homogeneous seawater, any particles were removed, the seawater was sterilized by filtering it through a 0.45 µm syringe filter, followed by sterilization. Filtration is one of the methods employed for sterilization and homogenization [13,14,37,38]. Seawater physicochemical characteristics are presented in Table 3. Supplementary Figures S1 and S2 show the UV–VIS spectra (Multiskan SkyHigh, Thermo Scientific, Cleveland, OH, USA) and vision of the blood agar of seawater before and after the filtration process. Metals were determined using inductively coupled plasma–optical emission spectrometry. Except for the total microorganism count, there was no significant difference between seawater and control seawater.

Various concentrations of endotoxin standards were obtained from Pierce Chromogenic Endotoxin Quant Kit (Thermo Fisher Scientific, Waltham, MA, USA) and prepared according to the guidance of the assay. Briefly, the endotoxin standard was reconstructed with endotoxin-free water at room temperature using the *E. coli* endotoxin standard vial to make 10 EU/mL endotoxin stock solution. The concentrations at 0.10 EU/mL, 0.50 EU/mL, and 2.00 EU/mL were prepared according to the product guidance using endotoxin-free water. These concentrations were selected according to the standard curve for high standard in the Endotoxin Quant Kit. Although the available studies in the field of endotoxin in seawater are limited, the studies showed that endotoxin concentrations span a wide range (not specified (<1) to 38,000 EU/mL) [14,15,39]. Therefore, we combined the analytical requirements of the endotoxin kit and literature information. Then, a specific dose of MNPs (1, 10, and 30 mg/mL) and various concentrations of endotoxin (0.10 EU/mL of endotoxin (C1), 0.50 EU/mL of endotoxin (C2), 2.00 EU/mL of endotoxin (C3)) were dispersed and mixed into seawater. The selected MNP doses mimic the occurrence of MNPs in various seawaters [40,41]. During the exposure processes, the seawater of each volume was gently stirred for 72 h. Then, MNPs were removed from the endotoxin- and MNPs-treated seawater. Seawater samples were also filtered using a 0.45 µm syringe filter. The removed MNPs were dried and stored for chemical characterization. The filtered seawater samples were analyzed for biochemical indicators, including biofilm formation, oxidative stress, and agglomeration. The controls were prepared by (i) only seawater control (filtered sample), (ii) no endotoxin control (seawater including only various MNPs), and (iii) no MNP control (seawater including various concentrations of endotoxins). The entire process, including MNP preparation, as well as endotoxin and MNP exposure, took place within a Class II biosafety cabinet to ensure a controlled environment (Nukleon NGK-120, Class II, Ankara, Türkiye, and Thermo Fisher Scientific, Safe 2020 Class II, Vantaa, Finland).

Seawater samples after incubation with endotoxin and MNPs were tested at the 200–650 nm wavelength using UV–VIS microplate spectrometry (Multiskan SkyHigh, Thermo Scientific, Cleveland, OH, USA).

The impact of the co-presence of endotoxin and MNPs on seawater agglomeration was examined using turbidimetry. This was assayed in the treated seawater and controls using UV–VIS microplate spectrometry (Multiskan SkyHigh, Thermo Scientific, Cleveland, OH, USA) at 600 nm.

The effect of endotoxin on the leaching of MNPs into seawater samples was analyzed using the humidification index (HI) in the seawater samples. HI levels were measured using the absorbance ratio at the wavelengths of 250 and 365 nm by UV–VIS spectrometry (Multiskan SkyHigh, Thermo Scientific, Cleveland, OH, USA) [24].

Structural characterization of MNPs after exposure to various concentrations of endotoxin was assessed using H-NMR (Agilent VNMRS 500 MHz, 11.74 T, Santa Clara, CA, USA), as previously described in the study of Baysal et al. [36].

Biofilm formation was assessed using crystal violet (CV) assay. After the exposure duration, the filtered seawater samples (200 μL) were transferred to round-bottomed 96-well plates. Then, crystal violet (CV) solution (0.01%, 50 μL) was added to the wells, dried for 15 min, and the excess CV was removed by washing with sterile water. Lastly, 125 μL of 95% ethanol was added to release fixed CV, and absorbances were measured at 595 nm (UV-VIS spectrometry, Multiskan SkyHigh, Thermo Scientific, Cleveland, OH, USA) [40,42].

Protein content in seawater samples was assayed using the Bradford method. Carbohydrate content in seawater samples was tested using the phenol sulfuric acid method, measured at 480 nm (UV-VIS spectrometry, Multiskan SkyHigh, Thermo Scientific, Cleveland, OH, USA) [33].

Oxidative characteristics were tested by the dithiothreitol (DTT) assay. Seawater samples and DTT (1 mM) were mixed and incubated for 30 min at 37  °C, then 5,50-dithiobis-(2-nitrobenzoic acid) was added and reacted with the remaining DTT. The samples were measured at 412 nm by a UV–VIS microplate spectrometer (Multiskan SkyHigh, Thermo Scientific, Cleveland, OH, USA).

Total antioxidant activities in seawater were investigated based on the CUPRAC method. In this method, samples mixed with 0.01 M CuCl_2_, 7.5 × 10^−3^ M neocuproine, and 1 M CH_3_COONH_4_ for 30 min and measured at 450 nm (UV-VIS spectrometry, Multiskan SkyHigh, Thermo Scientific, Cleveland, OH, USA) [33,43].

The differences between the control and the samples, as well as the differences among samples, were analyzed by ANOVA with a post hoc Tukey test (*p* < 0.05). SPPS 17.0 software (IBM, Armonk, NY, USA) was applied for the significance and Spearman correlation (two-tailed) tests.

## 5. Conclusions

We first demonstrated the effect of co-exposure of MNP and endotoxin on seawater biophysicochemical characteristics. The spectrometric analysis, including H-NMR and UV–VIS, revealed the interaction between endotoxin and MNPs in seawater. In addition to attachment of endotoxin onto MNPs, the turbidity, HI, and oxidative potential results were attributed to the leaching of MNPs into seawater due to the impact of the co-presence of endotoxin and MNPs. Biofilm formation and antioxidant capacity experiments in seawater revealed that the co-exposure of endotoxin and MNPs triggered biofilm formation and reduction of the antioxidant capacity of seawater, which was linked to the HI and oxidative indicators. Our results show that the occurrence of endotoxins owing to microbial contamination may change the physiological impact of MNPs on seawater.

## Figures and Tables

**Table 1:** Retention (%) of endotoxin in seawater with PET MNPs.

Exposure	MP1	MP2	MP3
Seawater-MP + EndoxC1	57.1 ± 2.6	2.3 ± 4.1	3.2 ± 1.8
Seawater-MP + EndoxC2	24.5 ± 3.3	9.1 ± 2.5	5.2 ± 4.1
Seawater-MP + EndoxC3	29.3 ± 4.9	7.8 ± 2.7	8.6 ± 2.3

**Table 2:** Normalized UV–VIS absorbances at 306 nm of treated and untreated seawater. RSD < 0.4%. EndoxC1: 0.10 EU/mL of endotoxin; EndoxC2: 0.50 EU/mL of endotoxin; EndoxC3: 2.00 EU/mL of endotoxin. PET MNP at the doses of 1 mg/mL (MP1), 10 mg/mL (MP2), and 30 mg/mL (MP3). (*) indicates significant difference with control-seawater; (**) indicates significant difference with control-MP + seawater; (***) indicates significant difference with control-Endox + seawater at the same endotoxin concentration.

Exposure	MP1	MP2	MP3
Control-Seawater	1.00	1.00	1.00
Control-Seawater + MP	0.99	0.98	1.00
Control-Seawater + EndoxC1	1.02 *	1.02 *	1.02
Control-Seawater + EndoxC2	0.95 *	0.95 *	0.95 *
Control-Seawater + EndoxC3	0.95 *	0.95 *	0.95 *
Seawater-MP + EndoxC1	0.91 *^,^**^,^***	1.20 *^,^**^,^***	0.99 ***
Seawater-MP + EndoxC2	0.87 *^,^**^,^***	0.91 *^,^**^,^***	1.09 *^,^**^,^***
Seawater-MP + EndoxC3	0.88 *^,^**^,^***	0.89 *^,^**^,^***	1.01 ***

**Table 3:** Physicochemical and microbiological characteristics of seawater.

Parameter	Seawater	Control Seawater (Filtered)
pH	7.48 ± 0.35	7.51 ± 0.27
Salinity, %	24.62 ± 1.63	25.43 ± 1.07
Dissolved O_2_, mg/L	8.93 ± 0.51	8.80 ± 0.43
Total suspended solids, mg/L	<LOD (<10)	<LOD (<10)
Total phosphorus, μg/L	<LOD (<50)	<LOD (<50)
Nitrite, μg/L	<LOD (<2)	<LOD (<2)
Ammonium, μg/L	<LOD (<10)	<LOD (<10)
Al, mg/L	9.3 ± 1.1	9.1 ± 0.78
Ca, mg/L	496 ± 29	512 ± 36
Cd, mg/L	<LOD (<0.003)	<LOD (<0.001)
Cr, mg/L	<LOD (<0.01)	<LOD (<0.01)
Cu, mg/L	4.3 ± 0.9	4.4 ± 0.3
Na, mg/L	14,905 ± 1208	13,897 ± 1907
Ni, mg/L	<LOD (<0.002)	<LOD (<0.002)
Mg, mg/L	1167 ± 225	1203 ± 341
Pb, mg/L	<LOD (<0.001)	<LOD (<0.001)
Zn, mg/L	17.8 ± 4.9	16.9 ± 3.8
Total microorganism count	238 ± 81	ND
Turbidity, a.u.	0.040 ± 0.002	0.042 ± 0.001
Protein, mg/mL	0.09 ± 0.12	0.28 ± 0.08
Carbohydrate, mg/mL	0.21 ± 0.09	0.34 ± 0.03
Antioxidant, µM Trolox	0.091 ± 0.006	0.086 ± 0.011

## Data Availability

Data is contained within the article or Supplementary Materials.

## Supplementary Materials

The following supporting information can be downloaded at: https://www.mdpi.com/article/10.3390/ijms26157178/s1.
