# Supplementary material for: Impact of Co-Presence of Endotoxins and Microplastics on Seawater Biophysicochemical Indicators
Source: Int J Mol Sci. 2025 Jul 25;26(15):7178. doi: 10.3390/ijms26157178 (PMC12346072; doi:10.3390/ijms26157178)

Supplementary Materials

Supplementary Figure S1. UV-VIS spectra of seawater before and after filtration.

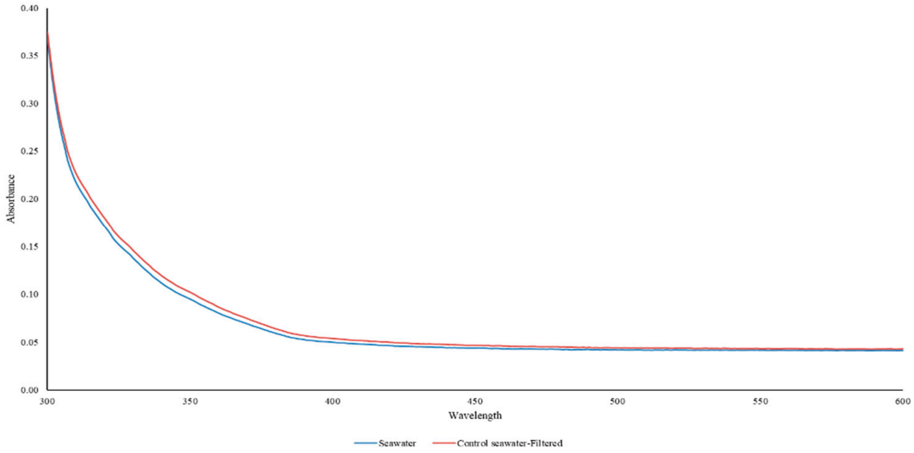

Supplementary Figure S2. Vision of the seawater and filtered seawater samples cultured on Blood agar.

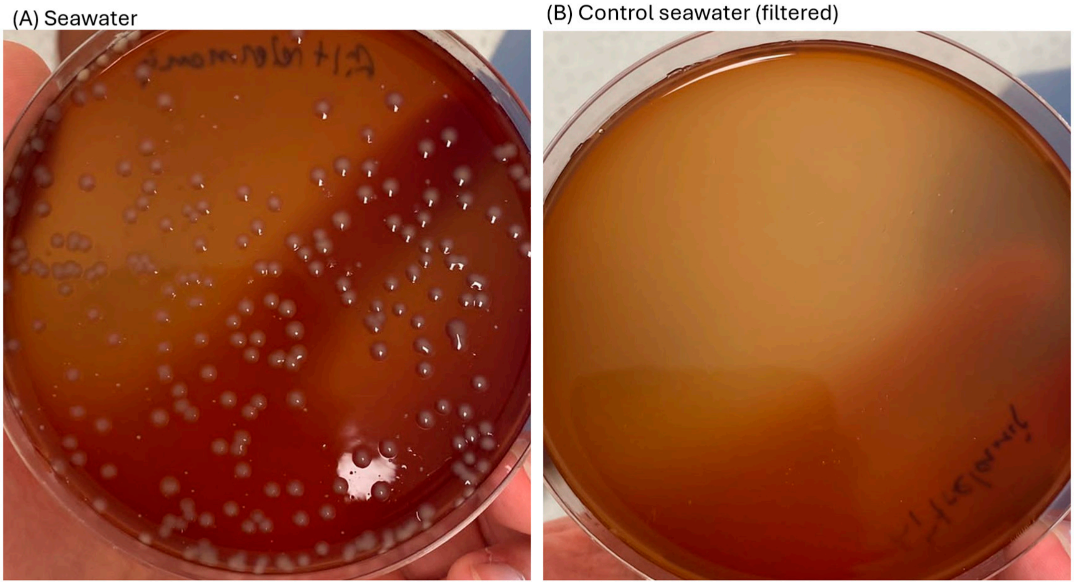

Supplement: Supplementary file 1 [file ijms-26-07178-s001.zip › ijms-3730931-supplementary.pdf]
